# The HuRaA Trial—The Radiocapitellar Line Shows Significant Posterior Translation in Healthy Elbows: A Prospective Analysis of 53 Healthy Individuals

**DOI:** 10.3390/biomedicines12122660

**Published:** 2024-11-21

**Authors:** Christian T. Schamberger, Tobias Grossner, Christoph Rehnitz, Sebastian Findeisen, Thomas Ferbert, Arnold J. Suda, Gerhard Schmidmaier, Stephan Stein

**Affiliations:** 1 Clinic for Trauma and Reconstructive Surgery, University Hospital Heidelberg, 69118 Heidelberg, Germany; 2 Diagnostic and Interventional Radiology, University Hospital Heidelberg, Im Neuenheimer Feld 420, 69120 Heidelberg, Germany; 3 Medical Faculty Mannheim, Ruprecht-Karls-University Heidelberg, 68167 Mannheim, Germany

**Keywords:** posterolateral rotatory instability, radiocapitellar line, radiocapitellar distance, MRI

## Abstract

**Background**: The elbow joint is stabilized by complex interactions between bony structures and soft tissues, notably the lateral and medial collateral ligaments. Posterolateral rotatory instability (PLRI), a form of elbow instability, is challenging to diagnose due to overlapping symptoms with other conditions. The radiocapitellar line (RCL) is a radiographic tool for assessing humeroradial alignment and elbow stability, but its diagnostic accuracy remains debated. This study aims to provide normative data on RCL deviations in healthy individuals to improve diagnostic criteria for PLRI. **Methods**: A prospective study was conducted with 53 healthy individuals (27 males, 26 females) aged 18–45 years. MRI scans of the participants’ elbows were performed in maximum extension and supination to assess radiocapitellar deviations (RCDs). Two orthopedic surgeons independently analyzed the images to evaluate RCDs and assess interobserver reliability. Statistical analyses, including independent *t*-tests and Pearson correlations, were used to explore the relationship between RCDs, demographic factors, and elbow stability. **Results**: The average RCD in the cohort was 1.77 mm (SD 1.06 mm). Notably, 62.9% of participants had deviations greater than 1.2 mm, while 12.9% exceeded 3.4 mm, thresholds traditionally used to diagnose PLRI. Gender and age did not significantly influence RCD values. The interobserver reliability was almost good (ICC = 0.87), supporting the consistency of the RCL measurements. **Conclusions**: Significant RCDs occur even in asymptomatic individuals, challenging the current diagnostic thresholds for PLRI based solely on RCL measurements. A comprehensive assessment that includes clinical, anatomical, and functional evaluations is essential for accurate diagnosis. These findings highlight the need for refined diagnostic criteria and further research into elbow stability.

## 1. Introduction

The elbow joint, a complex and highly mobile articulation, plays a crucial role in upper extremity function [1]. It facilitates a wide range of movements, including flexion, extension, pronation, and supination, enabling activities from daily tasks to high-performance sports. Understanding the biomechanics and stability of the elbow is essential for diagnosing and treating various conditions, including post-traumatic and degenerative disorders [2,3]. Assessing elbow stability is particularly challenging due to the intricate interplay of bony and soft tissue structures. In addition to the bony structures, the collateral ligaments are an important element of elbow stability. The lateral collateral ligament (LCL) and the medial collateral ligament (MCL) play pivotal roles in maintaining stability of the elbow [2,4,5]. Any disruption in this delicate balance can lead to instability, which may manifest as pain, reduced range of motion, and compromised functional abilities [2,6].

Posterolateral rotatory instability (PLRI) is one of the most recognized forms of elbow instability [6,7]. It typically arises from a traumatic event, such as a fall on an outstretched hand, chronic microtrauma, or as an iatrogenic complication of previous procedures including corticosteroid injections and debridement for lateral epicondylitis [6,8]. The diagnosis usually includes a detailed medical history with a focus on past injuries to the elbow followed by a clinical examination, in particular to assess elbow stability. If there are any indications of PLRI, sonographic assessment of the elbow and the soft tissues that can be visualized can be used. Further examination is then carried out using X-ray imaging and MR tomography to assess joint congruence and tendon and ligament structures. Clinically, PLRI is characterized by a combination of elbow pain, posterolateral rotatory instability, and in some cases restricted range of motion [6,9]. Diagnosis can be difficult, as symptoms may overlap with other conditions such as lateral epicondylitis or intra-articular findings like a symptomatic plica. Accurate diagnosis is crucial for effective treatment planning, which may range from conservative management to surgical intervention [6,10,11].

**Figure 1:**
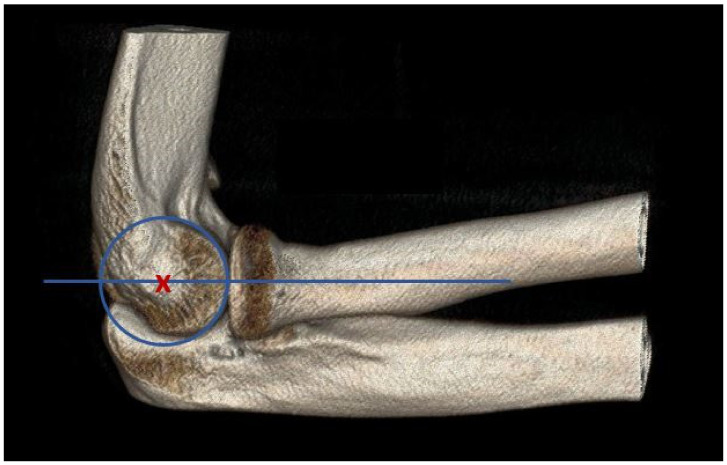
Three-dimensional reconstruction of an elbow showing the concept of the RCD. The red X represents the center of the capitulum. The blue line shows the axis of the radial neck, which lies exactly on the center of the capitulum.

The radiocapitellar distance (RCD) is used to assess joint congruence as a tool for assessing joint stability with the aid of the radiocapitellar line (RCL). The RCL has emerged as a valuable radiographic tool for assessing humeroradial alignment (HuRaA) and, consequently, elbow stability [12,13,14,15]. Traditionally, the RCD is determined using a lateral X-ray of the elbow, although in recent years, cross-sectional imaging such as MR and CT has also been used with the advantage that the soft tissue conditions can also be assessed directly. The RCL is defined as a line drawn from the center of the radial head to the center of the capitellum (Figure 1). Displacement of this line is believed to indicate instability, particularly in cases of PLRI. The current literature presents conflicting evidence regarding the diagnostic utility of the RCL [16,17,18]. Furthermore, thresholds are only occasionally mentioned in the literature, especially for evaluation via MRI. Some studies suggest that a deviation greater than 1.2 mm from the capitellar center may raise suspicion for instability, while others assert that deviations exceeding 3.4 mm confirm the diagnosis in MRI [13,14,15]. However, further studies claim that the use of RCL to assess instability is an unsafe procedure [16,18]. The discrepancies in these thresholds emphasize the need for a more nuanced understanding of RCL norms and its deviations in various populations. Most existing studies have focused only on patients with known instability, leaving a significant gap in knowledge regarding healthy individuals and therefore the critical clinical threshold [13,14,15].

This study aims to address this gap by investigating the RCL in a cohort of healthy individuals and employs a prospective design to evaluate the RCL in healthy elbows, aiming to generate data that can be used in future clinical practice for robust diagnosis or elbow instabilities. The decision was made to assess using MRI because it is a radiation-free and sufficient imaging method with high resolution and standardized examination procedures.

By analyzing MRI scans of participants in maximum extension and supination, the research will assess the degree of radiocapitellar deviation and explore its implications for understanding elbow stability. Ultimately, this study seeks to contribute to the ongoing discourse on elbow instability and the diagnostic utility of the RCL. By providing insights into the RCL in healthy individuals, this research may challenge existing diagnostic thresholds.

## 2. Material and Methods

### 2.1. Study Design

This prospective study was designed to evaluate the RCL in healthy individuals and to analyze its implications for diagnosing posterolateral rotatory instability (PLRI). Conducted according to the STROBE guidelines and the Declaration of Helsinki, it received ethical approval from the University of Heidelberg’s Medical Faculty (S-293/2024). Informed consent was obtained from all participants, and patient confidentiality was maintained. The study aimed to address the existing gaps in the literature regarding normative RCL measurements in asymptomatic populations.

### 2.2. Participants

A total of 53 healthy individuals were recruited for the study, comprising 27 males and 26 females, aged between 18 and 45 years. Participants were selected through advertisements and community outreach, ensuring a diverse representation of the population.

### 2.3. Inclusion Criteria

The inclusion criteria were as follows:

- Healthy individuals aged 18 to 45 years.
- No history of elbow injuries or surgeries.
- No clinical indications of elbow instability or pain.
- No musculoskeletal conditions affecting the upper extremities.
- Full range of motion.

### 2.4. Exclusion Criteria

The exclusion criteria were as follows:

- Previous trauma or surgery involving the elbow.
- Any history of elbow pain or dysfunction.
- Chronic conditions affecting joint stability or function, such as rheumatoid arthritis or other inflammatory disorders.

All participants underwent a thorough clinical assessment, including a detailed medical history and physical examination and assessment of the range of motion (ROM) to confirm their eligibility for the study. Participants who met the inclusion criteria were enrolled and scheduled for MRI examinations.

### 2.5. MRI Examination

To ensure optimal image quality, all subjects were imaged using a latest generation 3-Tesla MRI system with a 70 cm gantry width (Magnetom VIDA, Siemens Healthineers, Erlangen, Germany) and a high-resolution 18-channel flex coil. The sequence protocol included high-resolution T1-weighted turbo spin echo (TSE) images without fat saturation (repletion time–echo time (TE/TR) 484/9 ms, slice thickness 2.5 mm (in-plane resolution 0.28 mm), field of view 180 × 180 mm adjusted to capture the entire elbow joint, ensuring coverage of all relevant anatomical landmarks, imaging matrix 320) in axial, coronal, and sagittal orientations. Participants were positioned in the supine position, and the elbow was placed in maximum extension and supination to standardize the imaging protocol. This positioning was chosen to replicate the anatomical alignment typically seen during functional activities.

### 2.6. Radiocapitellar Line Assessment

**Figure 2:**
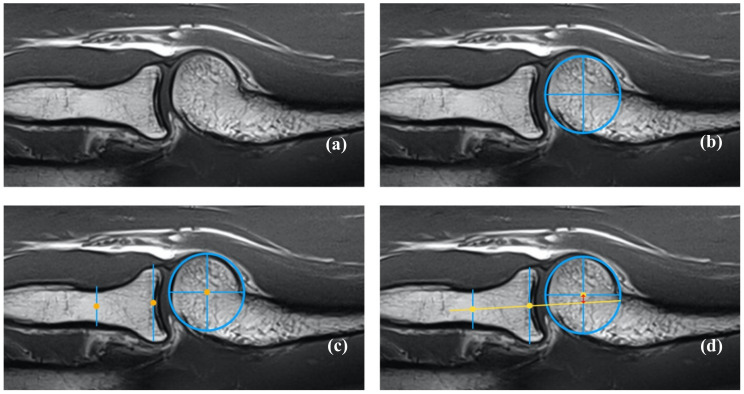
RCD measurement in MRI: (**a**) native MRI of the elbow in the sagittal section; (**b**) identification of the center of the capitulum; (**c**) identification of the radial neck axis; (**d**) drawing the RCL and measurement of the RCD.

Prior to assessing the MRI images with reference to the RCD, i.e., joint concurrence, the DICOM data set was checked for completeness, image quality, and correctness. The RCL was evaluated using standardized measurement techniques. Two orthopedic surgeons, one board-certified shoulder–elbow surgeon with extensive experience in musculoskeletal imaging (ultrasound instructor) and the other with less experience (resident), independently analyzed the MRI scans to assess the RCL. This dual assessment aimed to evaluate interobserver reliability and reduce potential bias (Figure 2).

The measurement procedure was performed as outlined below:

1. The identification of anatomical landmarks was carried out as follows:
  ○ The center of the capitellum was located at the most prominent point of the capitellar surface (Figure 2b).
  ○ The center of the radial head was identified as the midpoint of the radial head’s surface (Figure 2c).
  ○ The center of the radial neck was determined at the level of the supinator muscle (Figure 2c).
  ○ To validate the correct position, the corresponding locations were checked using a split screen and an orientation line in the transverse and coronal sections.
2. Drawing the RCL (yellow line) was carried out as follows:
  ○ A straight line was drawn connecting the center of the radial neck and the radial head to the center of the capitellum on the sagittal plane (Figure 2d).
3. Deviation measurements (red arrows) were determined as follows:
  ○ The distance from the capitellar center to the RCL was measured perpendicularly (Figure 2d).
  ○ Measurements were recorded in millimeters.
4. The thresholds for interpretation are outlined below:
  ○ A deviation of greater than 1.2 mm was considered suspicious for PLRI.
  ○ A deviation greater than 3.4 mm was regarded as confirming the diagnosis of elbow instability.

### 2.7. Statistical Analysis

Statistical analyses were performed using the Statistical Package for the Social Sciences (SPSS) software version 25.0 (IBM Corp., Armonk, NY, USA) to assess the data collected from the magnetic resonance imaging (MRI) measurements of the RCL. The analysis focused on summarizing the demographic characteristics of the participants and evaluating the RCD.

A power analysis was performed to estimate the required sample size. An effect size of Cohen’s d = 0.8 was assumed, which represents a large effect size and is frequently used in clinical studies. The significance level was set at 0.05 to keep the probability of an α error (false-positive finding) low. The aim of the analysis was to achieve a statistical power of 0.8, which means that the study has an 80% probability of detecting an existing effect. Based on these parameters, the necessary sample size was determined to ensure statistically meaningful results. The power analysis resulted in a sample size of at least 50 subjects [19].

Descriptive statistics were employed to summarize the demographic data, including age and gender distribution. Continuous variables were represented using means and standard deviations (SDs), while categorical variables were expressed in terms of frequencies and percentages.

To assess the proportion of elbows exhibiting deviations greater than the established diagnostic thresholds (1.2 mm and 3.4 mm) [13,14,15], the prevalence of significant posterior translation of the radial head was calculated. This prevalence was expressed as percentages of the total sample size, providing insight into the extent of potential instability within the cohort.

Interobserver reliability between the two orthopedic surgeons was assessed using intra-class correlation coefficients (ICCs). The ICC is a statistical measure that reflects how much of the variance in RCD measurements can be attributed to differences between observers, as opposed to measurement error. The criteria for interpreting ICC values were defined as follows: values ranging from 0.00 to 0.50 indicated poor agreement, 0.5 to 0.75 indicated moderate agreement, 0.76 to 0.90 indicated good agreement, and values greater than 0.90 indicated excellent agreement. A *p*-value of less than 0.05 was considered statistically significant [20].

Comparative analyses were performed using independent *t*-tests to evaluate differences in RCD measurements based on demographic variables such as gender and age. This comparative analysis aimed to identify any statistically significant differences in elbow stability measurements among various subgroups. Additionally, effect sizes were calculated using Cohen’s d to assess the magnitude of differences found in the RCD measurements between subgroups. An effect size of 0.2 was considered a small effect, 0.5 a medium effect, and 0.8 a large effect [19,21].

To investigate the relationship between gender as well as age and RCD measurements, Pearson correlation coefficients were calculated. This analysis aimed to determine whether age correlated with the magnitude of RCD deviations. The coefficients were interpreted based on established criteria, with values ranging from 0.00 to 0.19 indicating a very weak correlation, 0.20 to 0.39 a weak correlation, 0.40 to 0.59 a moderate correlation, 0.60 to 0.79 a strong correlation, and 0.80 to 1.00 indicating a very strong correlation. Furthermore, the ROM and the RCD were examined for correlations in a graphical comparative analysis [19,22].

The results were reported along with 95% confidence intervals (CIs) to provide a range of values within which the true population parameter is expected to lie. CIs were calculated for mean RCD values and proportions of cases exceeding the defined thresholds, offering additional context for the results. The significance level for all analyses was set at *p* ≤ 0.05, indicating the threshold at which the null hypothesis would be rejected [19].

## 3. Results

### 3.1. Demography

A total of 53 healthy individuals participated in the study, consisting of 27 males and 26 females. The mean age of the participants was 30 years, with an age range from 18 to 45 years. Demographic characteristics, including gender distribution, range of motion, and age, are summarized in Table 1.

### 3.2. Radiocapitellar Deviations

The primary outcome of the study was the evaluation of the RCD measured using MRI. The average RCD across all participants was found to be 1.77 mm, with a standard deviation of 1.06 mm. This indicates a significant amount of posterior translation of the radial head within the asymptomatic cohort (Table 2).

To assess the prevalence of significant deviations, we evaluated how many participants exceeded the established diagnostic thresholds for posterolateral rotatory instability (PLRI). The results revealed that 62.9% of the elbows exhibited deviations greater than the 1.2 mm threshold, which is considered suspicious for PLRI. Furthermore, 12,9% of the elbows showed deviations exceeding 3.4 mm, a measurement that confirms the diagnosis of elbow instability according to the current literature (Table 3).

### 3.3. Gender Differences

When analyzing the RCD based on gender, a comparative analysis using independent *t*-tests was performed. The average RCD for male participants was 1.8 mm (SD = 1.22 mm), while the average for female participants was 1.6 mm (SD = 00.87 mm). The independent *t*-test revealed no statistically significant difference between the two groups (*p* = 0.45), suggesting that gender does not significantly influence the RCD in this cohort.

### 3.4. Correlation of Range of Motion (ROM) and RCD

Both flexion (r = 0.114, *p* = 0.417) and extension (r = 0.121, *p* = 0.390) demonstrated very weak correlations (r < 0.19).

### 3.5. Age Correlation

The correlation between age and the RCD was also evaluated. The Pearson correlation coefficient indicated no statistically significant correlation (r = 0.012, *p* = 0.43) between age and the RCD measurements. This suggests that the relationship is not strong enough to draw definitive conclusions.

### 3.6. Interobserver Reliability

To assess the reliability of the RCD measurements, ICCs were calculated between the two orthopedic surgeons who analyzed the MRI scans. The ICC for the RCD measurements was found to be 0.87, indicating an almost good agreement between observers.

The results indicate that significant posterior translation of the radial head can occur even in asymptomatic individuals, raising important questions about the diagnostic criteria used for assessing elbow instability.

## 4. Discussion

This study suggests that the current diagnostic criteria for elbow instability, based on RCL deviations, may need to be revised. Furthermore, the findings of this study accent the complexity of elbow stability and the challenges in utilizing the radiocapitellar line (RCL) as a diagnostic tool for posterolateral rotatory instability (PLRI). With an average radiocapitellar deviation (RCD) of 1.77 mm in a cohort of healthy individuals, and in this study, 62.9% exceeding deviations greater than the 1.2 mm threshold and 12.9% showing deviations exceeding 3.4 mm, a value typically used to confirm elbow instability, it is evident that significant posterior translation of the radial head can occur in asymptomatic individuals [15]. No correlation between gender and RCD was found in our work. In addition, Gilotra et al. (2021) also found no significant differences between genders in their study on risk factors for atraumatic posterolateral rotational instability. This is in line with our results, suggesting that gender may not be a critical factor in this context [23].

There was also no statistically significant correlation between age and the RCD (r = 0.012, *p* = 0.43). However, this was to be expected, as the literature of recent years has not identified any age-related differences in relation to the RCD in adults; in fact, patients who have reached adulthood show a centering of the radial head between 16 and 18 years [24,25]. Zhou et al. [26] suggests that while age may influence certain orthopedic conditions, it does not significantly affect radiocapitellar alignment in adults. Tan et al. discussed the reliability of the radiocapitellar line in diagnosing elbow dislocations, noting that the alignment becomes more consistent after certain developmental milestones, but this does not imply a decline in alignment with aging in adults [24]. However, it should be noted that degenerative changes, especially osteoarthritis, can certainly have an influence on the biomechanics of the elbow joint, but an anterior translation of the radial head is found here [27]. In our healthy population, in the sense of the inclusion criteria, however, a dorsal translation was found.

The results of this prospective study support the findings and conclusions of retrospective studies conducted in recent years [16,17,18]. This raises critical questions about the reliability of RCL measurements in diagnosing elbow instability [13,15]. These findings challenge the reliability of current diagnostic criteria, as significant deviations were found in asymptomatic individuals.

Relying solely on RCL measurements for diagnosing PLRI could lead to both misdiagnosis and overdiagnosis, particularly in patients with no prior history of elbow injuries [18]. Given this, clinicians should interpret RCL deviations with caution. Isolated measurements may not be sufficient to diagnose PLRI accurately. Instead, a comprehensive diagnostic approach is needed, incorporating clinical assessments, patient history, and potentially other imaging modalities to achieve a more precise evaluation of elbow stability. Understanding the patient’s full clinical picture, rather than focusing solely on radiographic findings, will lead to better diagnostic accuracy and treatment outcomes.

Furthermore, the clinical presentation of PLRI is not limited to mechanical instability; it often involves symptoms such as pain, functional limitations, and reduced range of motion [6,8]. Thus, any diagnostic tool should account for both objective measurements, like RCL deviations, and subjective symptoms reported by the patient. Complementing RCL measurements with functional tests, such as the pivot shift test—a dynamic clinical evaluation of elbow stability—can provide a more holistic assessment of elbow function [28]. These functional tests can help determine the patient’s capacity to perform activities of daily living or engage in sports-specific movements, which are essential considerations in treatment planning. Ultimately, integrating clinical, anatomical, and functional assessments will enable more effective and tailored management of elbow instability conditions like PLRI.

The distinction between idiopathic and traumatic instability is essential for tailoring treatment strategies. Traumatic instability often necessitates immediate intervention, while idiopathic cases may benefit from a more conservative approach focused on rehabilitation and strengthening. Understanding the individual factors contributing to instability can guide personalized treatment plans [29,30,31].

The high interobserver reliability (ICC = 0.87) between the two orthopedic surgeons who independently assessed the MRI scans reinforces the reliability of the RCL as a measurement tool. This indicates that, when performed under standardized conditions, RCL measurements can produce consistent and reproducible results. However, relying solely on RCL deviations may not be sufficient for diagnosing PLRI, and other clinical and functional factors must be considered.

While this study provides valuable insights, several limitations must be acknowledged. Future studies should involve larger cohorts and more diverse populations to strengthen the validity of the results. Additionally, the reliance on MRI for measuring RCD presents potential measurement errors due to image resolution and interpretation variability. Standardized protocols and advanced imaging techniques may help mitigate these issues in future studies. The experience level of the interpreting surgeons may also introduce variability; thus, future research should consider using multiple reviewers to enhance reliability.

Future research should focus on developing more refined diagnostic thresholds for different populations and exploring the role of functional testing alongside imaging. Furthermore, longitudinal studies tracking healthy individuals with significant RCL deviations over time could help determine whether these deviations predispose an individual to instability later in life.

## 5. Conclusions

In summary, this study demonstrates that significant posterior translation of the radial head can occur in healthy individuals, challenging the current diagnostic thresholds for PLRI based solely on RCL measurements. The high prevalence of deviations greater than the established thresholds raises critical concerns regarding the use of the RCL as a standalone diagnostic criterion. These findings emphasize the importance of a multifaceted approach to elbow stability assessment that considers anatomical variability, functional factors, and a range of clinical evaluations. Future research should aim to establish more robust diagnostic criteria and improve understanding of the complex relationships between elbow anatomy, stability, and functional outcomes.

## Figures and Tables

**Table 1:** Demographic data of the study cohort.

	N	Percentage	Mean	SD
Sex				
Male	26	49.1		
Female	27	50.9		
Side				
Left	26	49.1		
Right	27	50.9		
Age			34	10.65
Range of Motion				
Flexion			145	6.61
Extension			6	4.74

**Table 2:** Results of the RCD measurements.

	N (Elbows)	RCD (mm)	SD
Sex			
Male	26	1.88	1.23
Female	27	1.67	0.88
Side			
Left	26	1.96	1.09
Right	27	1.59	1.01
Mean		1.77	1.06

**Table 3:** MR tomographically suspicious/confirmed PLRI.

	N (RCD ≥ 1.2 mm)	Percentage	N (RCD ≥ 3.4 mm)	Percentage
Sex				
Male	18	43	4	8
Female	16	30	1	2
Total	34	64	5	9
Side				
Left	15	28	2	4
Right	19	36	3	6
Total	34	64	5	9

## Data Availability

The data presented in this study are available on request from the corresponding author. The data are not publicly available due to privacy or ethical restrictions.
